# Data on dose-dependent cytotoxicity of rotenone and neuroprotection conferred by Yashtimadhu (*Glycyrrhiza glabra L.*) in an *in vitro* Parkinson's disease model

**DOI:** 10.1016/j.dib.2021.107535

**Published:** 2021-11-06

**Authors:** Gayathree Karthikkeyan, Ashwini Prabhu, Ravishankar Pervaje, Sameera Krishna Pervaje, Prashant Kumar Modi, Thottethodi Subrahmanya Keshava Prasad

**Affiliations:** a Center for Systems Biology and Molecular Medicine, Yenepoya Research Centre, Yenepoya (Deemed to be University), Mangalore 575018, India; b Yenepoya Research Centre, Yenepoya (Deemed to be University), Mangalore 575018, India; c Sushrutha Ayurveda Hospital, Puttur 574201, India; d Yenepoya Medical College and Hospital, Yenepoya (Deemed to be University), Mangalore 575018, India

**Keywords:** Neurodegeneration, Complementary and alternative medicine, Ayurveda, Actionable molecules

## Abstract

The data described in this article presents the toxicity of rotenone and the neuroprotective effect of Yashtimadhu choorna (powder) in an *in vitro* Parkinson's disease model [1]. Yashtimadhu choorna is prepared from the roots of *Glycyrrhiza glabra L.*, commonly known as licorice/ liquorice. The effects of rotenone and Yashtimadhu was assessed using cellular and molecular assays such as cell cytotoxicity assay, live-dead cell staining assay, cell cycle analysis, and western blotting. Protein-protein interaction was studied using ANAT plug-in in Cytoscape. Rotenone displayed time and dose-dependent toxicity, as evidenced by cell cytotoxicity assay and live-dead cell staining assay. Yashtimadhu showed no toxicity and prevented rotenone-induced toxicity. Rotenone and Yashtimadhu displayed differential control on the cell cycle. The Protein-interaction network showed the proteins interacting with ERK-1/2 and the pathways regulated by these interactions. The pathways regulated were primarily involved in cellular oxidative stress and apoptosis response. The data described here will enable the extent of cellular toxicity as a result of rotenone treatment and the neuroprotection conferred by Yashtimadhu choorna. This will enable understanding and exploring the effect of traditional and complementary medicine and aiding the identification of molecular targets to confer neuroprotection in Parkinson's disease.

## Specifications Table

**Table:** 

Subject	Biological sciences Neuroscience: Cellular and Molecular
Specific subject area	Cell and molecular biology
Type of data	Table Image Graph Figure
How data were acquired	Cytotoxicity data was acquired from IMR-32 cells treated with rotenone and Yashtimadhu extract (*Glycyrrhiza glabra L.*) using BMG LABTECH FLUOstar Omega multidetection microplate reader. Cell cycle analysis data was acquired from Guava® easyCyte Flow Cytometer and processed using FCS Express software, version 6. Live-dead assay and ROS assay cell imaging were carried out using ZOE Fluorescent Cell Imager, Bio-Rad, and data processed using ImageJ. Western blotting data were analyzed using ImageJ software. Statistical analysis using GraphPad Prism 5.
Data format	Raw and analyzed data
Parameters for data collection	Parameters for the data collection were as follows: IMR-32 cells were seeded at different densities based on the cell culture plate; 96-well plate: 5000 cells/well, 12-well plate: 10,000 cells/well, and 6-well plate: 30,000 cells/ well. Cells were differentiated with 10 µM retinoic acid for 9 days and treated with different concentrations of rotenone and Yashtimadhu (*Glycyrrhiza glabra L.*) aqueous extract.
Description of data collection	Treated cells were assayed for cytotoxicity using MTT reagent, live-dead cell staining assay using propidium iodide and HOECHST stains, ROS assay was carried out with cell staining using 2′,7′ –dichlorofluorescin diacetate and HOECHST stains, cell cycle analysis was carried out using propidium iodide staining, and protein expression was assayed using western blotting analysis. Protein interaction analysis using ANAT plug-in of Cytoscape version 3.8.
Data source location	Institution: Center for Systems Biology and Molecular Medicine, Yenepoya Research Centre, Yenepoya (Deemed to be University) City/Town/Region: Mangalore, Karnataka Country: India Latitude and longitude for collected samples/data: 12.8113° N, 74.8814° E
Data accessibility	With the article
Related research article	Karthikkeyan, G., Pervaje, R., Pervaje, S. K., Prasad, T.S.K., Modi, P.K. Prevention of MEK-ERK-1/2 hyper-activation underlines the neuroprotective effect of *Glycyrrhiza glabra L.* (Yashtimadhu) against rotenone-induced cellular and molecular aberrations. J Ethnopharmacol 274 (2021)114025.

## Value of the Data

• The data described here explains the effect of Yashtimadhu (*Glycyrrhiza glabra L.*) extract and rotenone on neuronal differentiated IMR-32 cells.
• The data also describes the dose-dependent effect of rotenone and the extent of the resulting cellular damage.
• The data will also be useful for researchers working on the effect of traditional and complementary medicine and for researchers working on Parkinson's disease (PD) model.
• The protein-protein interactions and the pathways regulated by the proteins regulated by Yashtimadu can offer newer insights to understanding its effect in an *in vitro* PD model.
• The dataset can be used to explore the neuroprotective mechanism of traditional and complementary medicine.
• The dataset can be used to identify molecular targets that can confer neuroprotection in PD.

## 1 Data Description

The data in this article depicts the effect of rotenone and Yashtimadhu extract (*Glycyrrhiza glabra L.*) on differentiated IMR-32 cells in the context of neuroprotection conferred by Yashtimadhu in the Parkinson's disease model [1]. Parkinson's disease is the selective loss of dopaminergic neurons, attributable to mitochondrial dysfunction and oxidative stress [2]. Rotenone is a mitochondrial complex-I inhibitor, which leads to the loss of the mitochondrial membrane potential, oxidative stress, and thereby inducing apoptosis [3]. Indian Ayurvedic system classifies plants with neuroprotective properties as *Medhya Rasayana*, which includes, Yashtimadhu, *Glycyrrhiza glabra*; Mandukaparni, *Centella asiatica* and, Guduchi, *Tinospora cordifolia* [4,5]. Yashtimadhu powder is prepared from licorice root is known for its potential as neuroprotective, memory enhancer, and immunomodulatory effects [5], [6], [7], [8]. The data explored the cellular toxicity of rotenone and neuroprotection by Yashtimadhu.

Cell cytotoxicity assay was used to determine the treatment concentrations of rotenone and Yashtimadhu extract. Yashtimadhu aqueous extract and rotenone dissolved in DMSO were used to test their effects on IMR-32 cells. Upon treatment with different concentrations of rotenone for 24 h and 48 h, the cells displayed a time- and dose-dependent toxicity. The absorbance and the percentage of cell death estimated with respect to control are shown in Tables 1 and 2.

**Table 1:** Cell cytotoxicity analysis with rotenone treatment for 24 h.

Sample	Replicate	560 nm	650 nm	Background subtraction	Blank subtraction	% cell viability	Average % cell viability
Blank	Rep_1	0.036	0.031	0.005			
	Rep_2	0.034	0.032	0.002	0	NA	NA
	Rep_3	0.042	0.039	0.003			
Ctrl	Rep_1	0.93	0.163	0.767	0.762	100.00	100.00
	Rep_2	0.988	0.19	0.798	0.796	100.00	
	Rep_3	0.921	0.174	0.747	0.744	100.00	
DMSO	Rep_1	1.008	0.205	0.803	0.798	104.72	96.69
	Rep_2	0.892	0.169	0.723	0.721	90.58	
	Rep_3	0.832	0.124	0.708	0.705	94.76	
0.25 nM	Rep_1	0.92	0.219	0.701	0.696	91.34	87.50
	Rep_2	0.883	0.167	0.716	0.714	89.70	
	Rep_3	0.734	0.125	0.609	0.606	81.45	
0.5 nM	Rep_1	0.792	0.109	0.683	0.678	88.98	88.78
	Rep_2	0.829	0.148	0.681	0.679	85.30	
	Rep_3	0.801	0.113	0.688	0.685	92.07	
1 nM	Rep_1	0.66	0.089	0.571	0.566	74.28	80.50
	Rep_2	0.674	0.091	0.583	0.581	72.99	
	Rep_3	0.821	0.117	0.704	0.701	94.22	
10 nM	Rep_1	0.623	0.097	0.526	0.521	68.37	77.23
	Rep_2	0.694	0.097	0.597	0.595	74.75	
	Rep_3	0.815	0.153	0.662	0.659	88.58	
100 nM	Rep_1	0.608	0.101	0.507	0.502	65.88	67.77
	Rep_2	0.675	0.145	0.53	0.528	66.33	
	Rep_3	0.621	0.089	0.532	0.529	71.10	
200 nM	Rep_1	0.616	0.166	0.45	0.445	58.40	60.18
	Rep_2	0.527	0.109	0.418	0.416	52.26	
	Rep_3	0.696	0.173	0.523	0.52	69.89	
500 nM	Rep_1	0.564	0.117	0.447	0.442	58.01	57.71
	Rep_2	0.552	0.1	0.452	0.45	56.53	
	Rep_3	0.538	0.099	0.439	0.436	58.60	
1 µM	Rep_1	0.723	0.219	0.504	0.499	65.49	59.26
	Rep_2	0.551	0.129	0.422	0.42	52.76	
	Rep_3	0.573	0.127	0.446	0.443	59.54	
10 µM	Rep_1	0.54	0.118	0.422	0.417	54.72	54.41
	Rep_2	0.507	0.082	0.425	0.423	53.14	
	Rep_3	0.522	0.107	0.415	0.412	55.38

**Table 2:** Cell cytotoxicity analysis with rotenone treatment for 48 h.

Sample	Replicate	560 nm	650 nm	Background subtraction	Blank subtraction	% cell viability	Average % cell viability
Blank	Rep_1	0.038	0.03	0.008			
	Rep_2	0.037	0.029	0.008	0	NA	NA
	Rep_3	0.032	0.03	0.002			
Ctrl	Rep_1	0.658	0.15	0.508	0.5	100.00	100.00
	Rep_2	0.682	0.108	0.574	0.566	100.00	
	Rep_3	0.722	0.13	0.592	0.59	100.00	
DMSO	Rep_1	0.605	0.117	0.488	0.48	96.00	105.47
	Rep_2	0.742	0.111	0.631	0.623	110.07	
	Rep_3	0.762	0.109	0.653	0.651	110.34	
0.25 nM	Rep_1	0.58	0.106	0.474	0.466	93.20	84.90
	Rep_2	0.534	0.08	0.454	0.446	78.80	
	Rep_3	0.579	0.089	0.49	0.488	82.71	
0.5 nM	Rep_1	0.505	0.082	0.423	0.415	83.00	73.33
	Rep_2	0.473	0.08	0.393	0.385	68.02	
	Rep_3	0.494	0.085	0.409	0.407	68.98	
1 nM	Rep_1	0.511	0.089	0.422	0.414	82.80	66.66
	Rep_2	0.423	0.077	0.346	0.338	59.72	
	Rep_3	0.424	0.083	0.341	0.339	57.46	
10 nM	Rep_1	0.377	0.07	0.307	0.299	59.80	62.78
	Rep_2	0.448	0.076	0.372	0.364	64.31	
	Rep_3	0.457	0.076	0.381	0.379	64.24	
100 nM	Rep_1	0.34	0.082	0.258	0.25	50.00	45.88
	Rep_2	0.353	0.085	0.268	0.26	45.94	
	Rep_3	0.346	0.098	0.248	0.246	41.69	
200 nM	Rep_1	0.308	0.087	0.221	0.213	42.60	38.03
	Rep_2	0.289	0.073	0.216	0.208	36.75	
	Rep_3	0.271	0.064	0.207	0.205	34.75	
500 nM	Rep_1	0.237	0.076	0.161	0.153	30.60	30.73
	Rep_2	0.225	0.067	0.158	0.15	26.50	
	Rep_3	0.331	0.122	0.209	0.207	35.08	
1 µM	Rep_1	0.244	0.075	0.169	0.161	32.20	27.58
	Rep_2	0.236	0.081	0.155	0.147	25.97	
	Rep_3	0.212	0.065	0.147	0.145	24.58	
10 µM	Rep_1	0.223	0.073	0.15	0.142	28.40	25.10
	Rep_2	0.209	0.068	0.141	0.133	23.50	
	Rep_3	0.214	0.074	0.14	0.138	23.39	

**Fig. 1:**
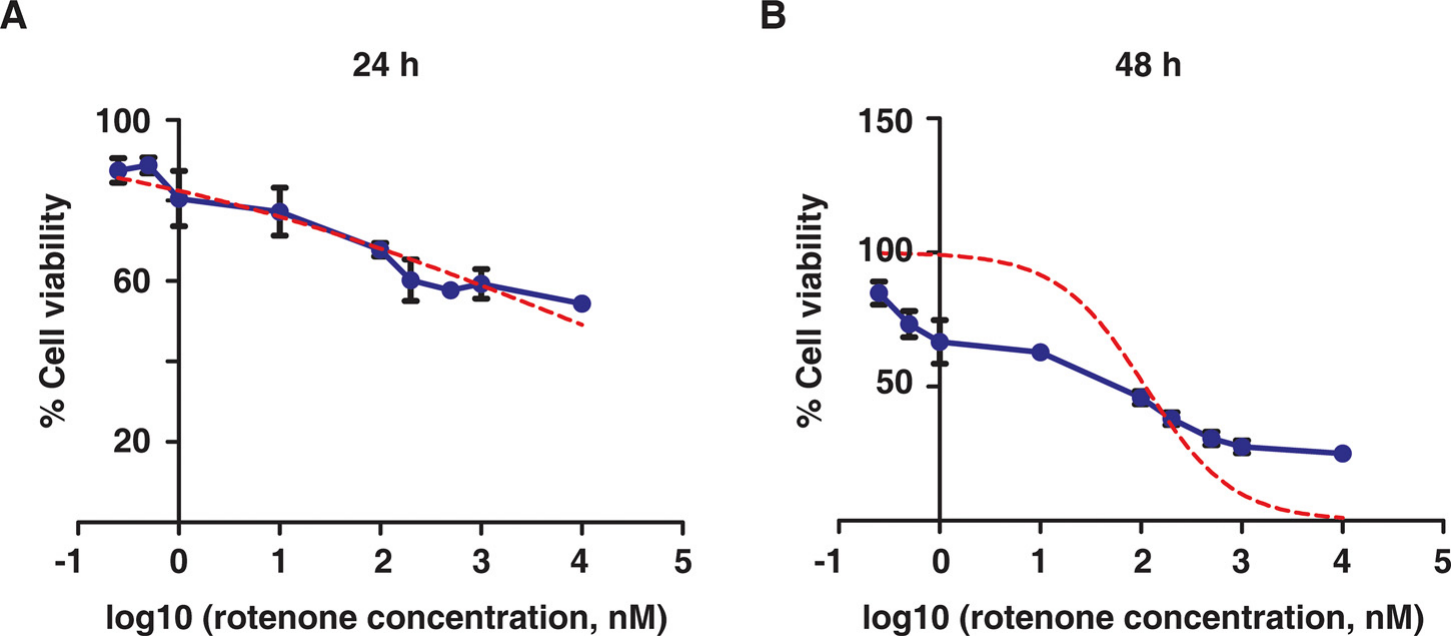
The Dose-response curve of cell viability with respect to treatment with different concentrations of rotenone. (A) Rotenone treatment for 24 h, (B) Rotenone treatment for 48 h. The red-line indicates the normalized dose-response curve-fit, and the blue-line represents the observed cell viability with the respective concentrations.

Non-linear curve fitting with the dose-response with respect to rotenone concentration was used for calculating the IC_50_, using the GraphPad Prism software. The IC_50_ concentration of rotenone was found to be 8.1 µM and 110 nM for 24 h and 48 h, respectively (Fig. 1).

The treatment of the IMR-32 cells with Yashtimadhu extract did not show any toxicity, and the absorbance observed using the cytotoxicity assay and the cell viability for the different concentrations of Yashtimadhu, are given in Table 3.

**Table 3:** Cell cytotoxicity assay with Yashtimadhu extract treatment for 48 h.

Sample	Replicate	560 nm	630 nm	Background subtraction	Blank subtraction	% cell viability	Average % cell viability
Blank	Rep_1	0.046	0.0325	0.0135			
	Rep_2	0.044	0.031	0.013	0	NA	NA
	Rep_3	0.046	0.0335	0.0125			
Control	Rep_1	0.736	0.1225	0.6135	0.6	100.00	100.00
	Rep_2	0.7575	0.1235	0.634	0.621	100.00	
	Rep_3	0.605	0.1005	0.5045	0.492	100.00	
50 µg/ml	Rep_1	0.737	0.1275	0.6095	0.596	99.33	98.13
	Rep_2	0.7775	0.13	0.6475	0.6345	102.17	
	Rep_3	0.592	0.1225	0.4695	0.457	92.89	
100 µg/ml	Rep_1	0.762	0.1175	0.6445	0.631	105.17	98.65
	Rep_2	0.807	0.145	0.662	0.649	104.51	
	Rep_3	0.5295	0.0925	0.437	0.4245	86.28	
200 µg/ml	Rep_1	0.665	0.1155	0.5495	0.536	89.33	99.18
	Rep_2	0.82	0.149	0.671	0.658	105.96	
	Rep_3	0.622	0.1065	0.5155	0.503	102.24	
500 µg/ml	Rep_1	0.7235	0.152	0.5715	0.558	93.00	98.76
	Rep_2	0.7655	0.1565	0.609	0.596	95.97	
	Rep_3	0.6855	0.145	0.5405	0.528	107.32	
1000 µg/ml	Rep_1	0.598	0.1225	0.4755	0.462	77.00	78.98
	Rep_2	0.635	0.1235	0.5115	0.4985	80.27	
	Rep_3	0.514	0.1095	0.4045	0.392	79.67	
1500 µg/ml	Rep_1	0.722	0.1435	0.5785	0.565	94.17	84.31
	Rep_2	0.56	0.1145	0.4455	0.4325	69.65	
	Rep_3	0.549	0.098	0.451	0.4385	89.13	

The cells were also co-treated with Yashtimadhu extract at 200 µg/ml, and two different concentrations of rotenone, 50 nM, and 100 nM. The co-treatment with Yashtimadhu showed lesser toxicity in comparison with the treatment of respective rotenone concentrations (Tables 4 and 5).

**Table 4:** Cell cytotoxicity assay with 50 nM rotenone and 200 µg/ml Yashtimadhu extract co-treatment for 48 h.

Sample	Replicate	560 nm	630 nm	Background subtraction	Blank subtraction	% cell viability	Average % cell viability
Blank	Rep_1	0.0427	0.0303	0.0123			
	Rep_2	0.0383	0.0303	0.0080	0	NA	NA
	Rep_3	0.0383	0.0303	0.0080			
Control	Rep_1	1.0713	0.2233	0.8480	0.8357	100.00	100.00
	Rep_2	1.1007	0.1610	0.9397	0.9317	100.00	
	Rep_3	1.1053	0.1633	0.9420	0.9340	100.00	
Rotenone 50 nM	Rep_1	0.8130	0.1050	0.7080	0.6957	83.25	76.48
	Rep_2	0.8000	0.1053	0.6947	0.6867	73.70	
	Rep_3	0.7993	0.1143	0.6850	0.6770	72.48	
Rotenone 50 nM + Yashtimadhu 200 µg/ml	Rep_1	0.9323	0.1803	0.7520	0.7397	88.51	86.08
	Rep_2	0.9297	0.1570	0.7727	0.7647	82.08	
	Rep_3	0.9800	0.1533	0.8267	0.8187	87.65	
Yashtimadhu 200 µg/ml	Rep_1	1.0863	0.1717	0.9147	0.9023	107.98	103.09
	Rep_2	1.0157	0.1450	0.8707	0.8627	92.59	
	Rep_3	1.1733	0.1500	1.0233	1.0153	108.71

**Table 5:** Cell cytotoxicity assay with 100 nM rotenone and 200 µg/ml Yashtimadhu extract co-treatment for 48 h.

Sample	Replicate	560 nm	630 nm	Background subtraction	Blank subtraction	% cell viability	Average % cell viability
Blank	Rep_1	0.0427	0.0303	0.0123			
	Rep_2	0.0564	0.0455	0.0109	0	NA	NA
	Rep_3	0.0557	0.0439	0.0118			
Control	Rep_1	1.0943	0.2040	0.8903	0.8780	100.00	100.00
	Rep_2	2.2680	0.2322	2.0358	2.0249	100.00	
	Rep_3	2.3037	0.2362	2.0675	2.0557	100.00	
Rotenone 100nM	Rep_1	0.6525	0.1000	0.5525	0.5402	61.52	53.93
	Rep_2	1.1915	0.1546	1.0369	1.0260	50.67	
	Rep_3	1.1709	0.1396	1.0314	1.0196	49.60	
Rotenone 100 nM + Yashtimadhu 200 µg/ml	Rep_1	0.8633	0.1557	0.7077	0.6953	79.20	74.76
	Rep_2	1.8263	0.1956	1.6307	1.6198	79.99	
	Rep_3	1.5189	0.1692	1.3497	1.3379	65.08	
Yashtimadhu 200 µg/ml	Rep_1	1.0833	0.1617	0.9217	0.9093	103.57	109.89
	Rep_2	2.5736	0.2595	2.3141	2.3032	113.74	
	Rep_3	2.5775	0.2560	2.3216	2.3098	112.36	

**Fig. 2:**
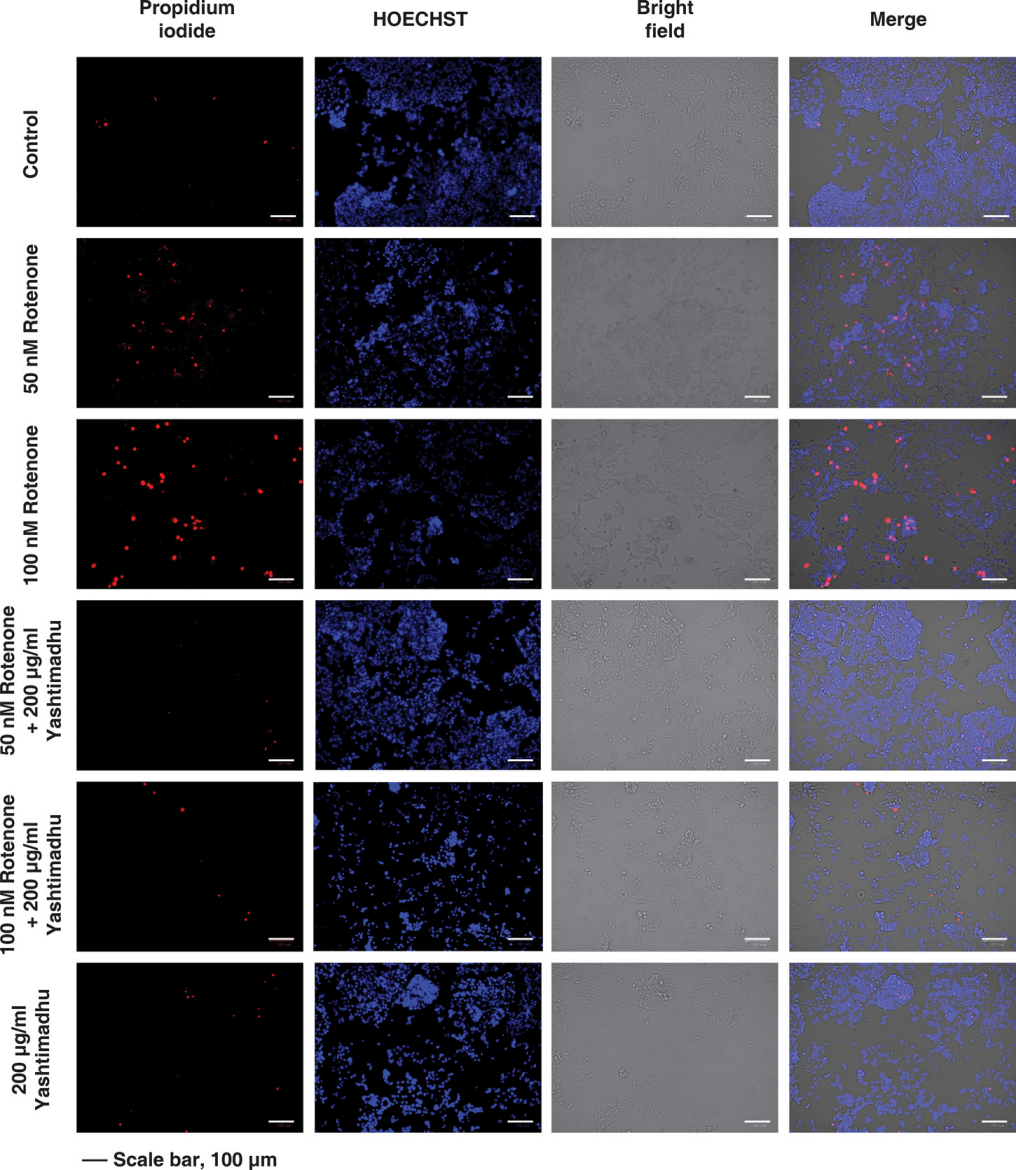
Live/dead cell staining assay using propidium iodide (red) to stain dead cells and HOECHST nuclear stain as a counterstain. Cells were treated with different concentrations of rotenone, 50 nM and 100 nM, and 200 µg/ml Yashtimadhu extract. The 50 nM and 100 nM rotenone treatment resulted in a significant (*p* < 0.05) reduction in live cells compared to control, and the rotenone+Yashtimadhu co-treatment resulted in a significant (*p* < 0.05) restoration of the live cells compared to the rotenone alone treatment groups.

In addition to the cell cytotoxicity assay, a live-dead staining assay was also carried out on the differentiated IMR-32 cells to evaluate the effect of rotenone at 50 nM and 100 nM concentrations and the protective effect of Yashtimadhu extract at 200 µg/ml concentration (Fig. 2).

**Fig. 3:**
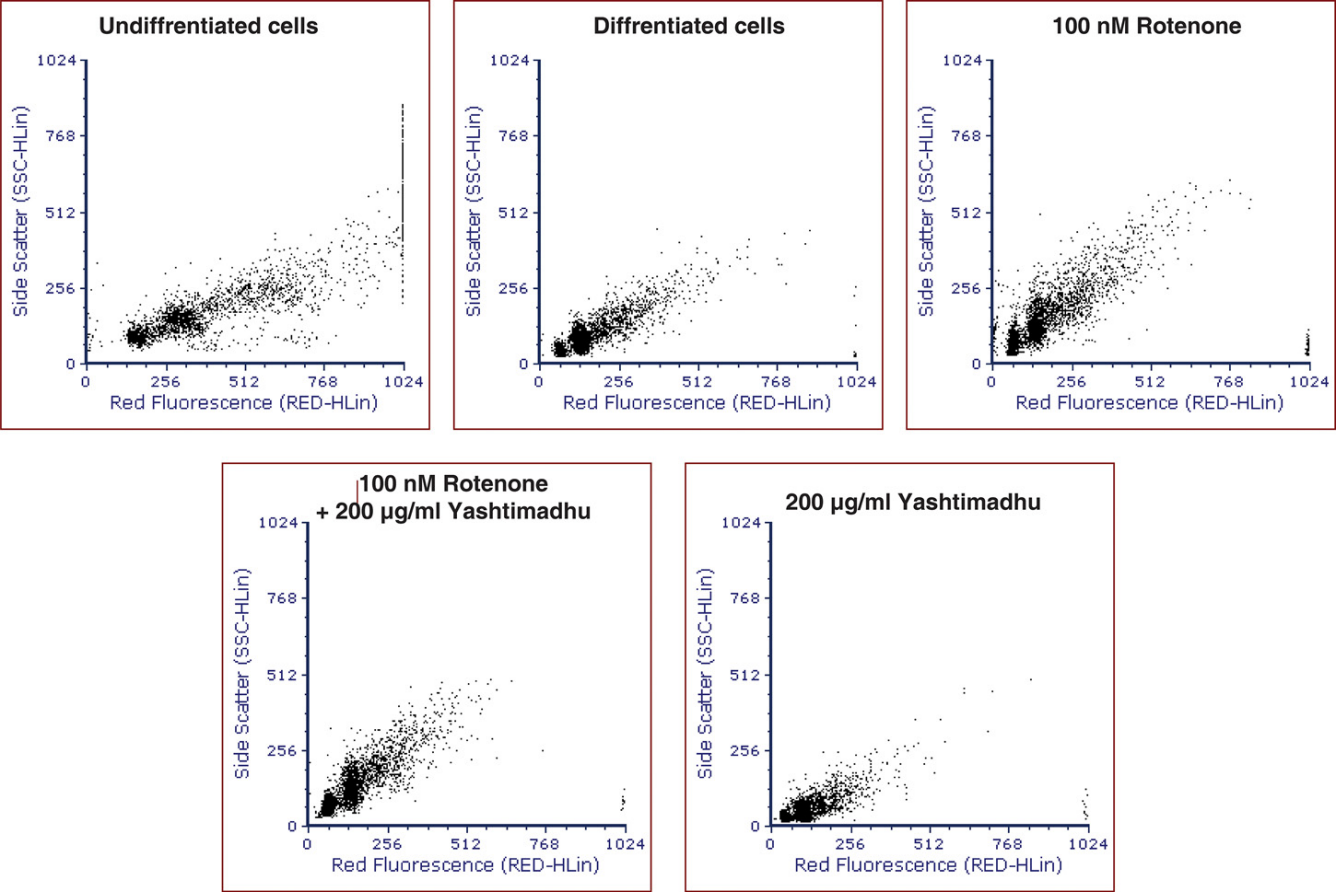
Scatter plot of cell cycle analysis of undifferentiated cells, and the differentiated cells treated with rotenone, rotenone + Yashtimadhu co-treatment and Yashtimadhu alone, which was assessed after 48 h of treatment.

The differentiated IMR-32 cells were treated with 100 nM rotenone and 200 µg/ml Yashtimadhu extract to analyze the effect of the treatments on cell cycle progression. The undifferentiated cells were also subject to cell cycle analysis. The scatter plots from the treatments are given in Fig. 3, and the percentage of cells in each of the cell cycle phases, such as G0/G1 (Population of cells in Go and G1 phases of cell cycle), S (population of cells in Synthesis phase), and G2/M (Population of cells in G2 and mitotic phases of cell cycle), is given in Table 6 and the raw FCS files are provided as supplementary files.

**Table 6:** Table showing the percentage of cells in different phases of cell cycle assessed after 48 h treatment.

Sample	Replicate	% G0/G1	% S	% G2/M	Average % G0/G1	Average % S	Average % G2/M
Un-differentiated cells	Rep_1	52.59	32.01	15.39	56.93	28.95	14.13
	Rep_2	61.26	25.88	12.86			
	Rep_3	62.86	25.88	12.86			
Control	Rep_1	68.89	24.53	6.58	56.13	38.84	5.03
	Rep_2	50.13	46.21	3.66			
	Rep_3	49.36	45.78	4.86			
Rotenone 100nM	Rep_1	44.94	27.40	27.66	40.28	21.24	38.49
	Rep_2	46.65	19.12	34.23			
	Rep_3	29.24	17.19	53.57			
Rotenone 100 nM + Yashtimadhu200 µg/ml	Rep_1	60.89	28.79	10.33	57.72	36.50	5.79
	Rep_2	55.76	37.20	7.04			
	Rep_3	56.50	43.50	0.00			
Yashtimadhu 200 µg/ml	Rep_1	52.54	46.44	1.03	53.39	45.08	1.53
	Rep_2	62.94	33.50	3.56			
	Rep_3	44.70	55.30	0.00			

### 1.1 Protein expression and interaction

Rotenone showed the IC_50_ at 100 nM therefore, the 100 nM rotenone concentration was used to assess the neuroprotective effect of Yashtimadhu.

**Fig. 4:**
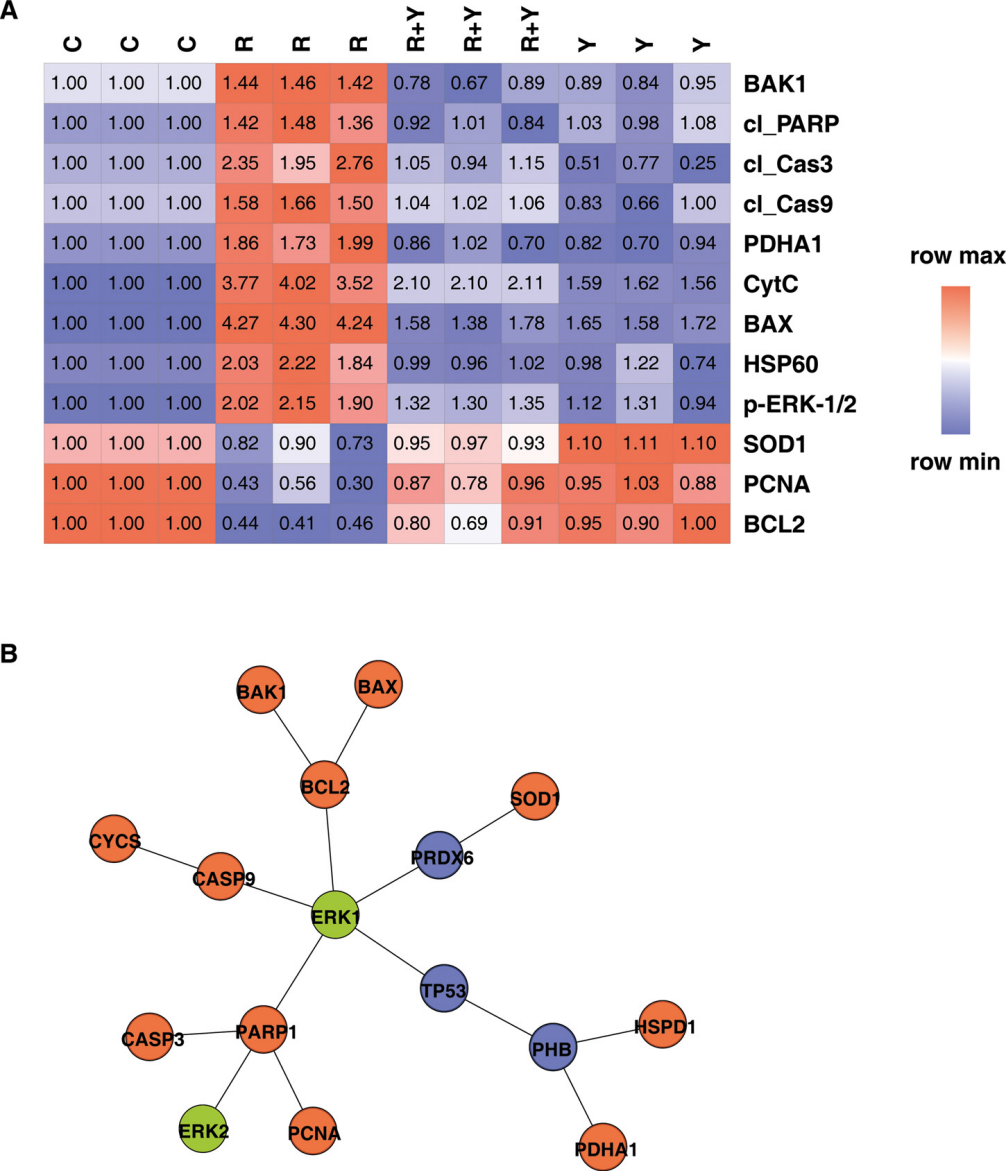
*Protein expression and Protein-protein interaction map***.** (A) Heat map giving the protein expression across the replicates and groups,which were significantly (*p* < 0.05) altered between rotenone vs control, all of the proteins were significantly restored by Yashtimadhu (*p* < 0.05) co-treatment, for 48 h treatment time, (B) Protein-protein interaction. Kinase ERK-1/2 is displayed in green, and the proteins studied for their expression in orange, while the intermediary interaction nodes are given in blue, which were not studied for their expression. Legends: C: Control, R: 100 nM rotenone, R+Y: Rotenone 100 nM + Yashtimadhu 200 µg/ml**,** Y: Yashtimadhu 200 µg/ml**.**

The expression of proteins was analyzed using western blotting and their fold change of expression was calculated with respect to untreated control cells. The fold change values are presented as a heat map with the values in the boxes representing each replicate from the respective groups, Fig. 4A. The interaction network of these proteins is given in Fig. 4B, and the significance of their interaction is given in Table 7.

**Table 7:** Pathways regulated by the protein-protein interaction that were altered with rotenone and Yashtimadhu, after 48 h treatment.

Interaction nodes	Confidence	Gene Ontology annotation	p-value	Corrected p-value	KEGG annotation	p-value	Corrected p-value
ERK1/2, PARP1, CASP3	0.96	Cellular response to chemical stress	3.0E-06	1.8E-03	Apoptosis	5E-06	4.7E-04
ERK1, TP53, PHB, PDHA1	0.84	Regulation of protein stability	1.3E-05	3.7E-03	Central carbon metabolism in cancer	6.6E-07	2.0E-04
ERK2, PARP1, ERK1, TP53, PHB, PDHA1	0.78	Cellular response to oxidative stress	3.2E-07	4.1E-04	Central carbon metabolism in cancer	2.8E-08	3.5E-05
ERK2, PARP1, ERK1, TP53, PHB, HSPD1	0.78	Response to oxidative stress	1.7E-08	1.3E-04	Apoptosis	4.1E-07	1.5E-04
ERK1/2, PARP1, PCNA	0.96	Cellular response to oxidative stress	1.8E-06	1.3E-03	Base excision repair	5E-05	2.0E-03
ERK1, PARP1, PCNA	0.96	Cellular response to oxidative stress	1.8E-06	1.3E-03	Base excision repair	5E-05	2.0E-03
ERK1/2, PARP1	0.96	Cellular response to metal ion	9.5E-05	1.3E-02	Apoptosis	2.9E-04	6.1E-03

## 2 Experimental Design, Materials and Methods

### 2.1 Materials

Propidium iodide, rotenone, retinoic acid, HOECHST-33342, MTT (3-(4,5-dimethylthiazol-2-yl)-2,5-diphenyltetrazolium-bromide), 2′,7′-Dichlorofluorescein diacetate (DCFDA), and collagen were procured from Sigma-Aldrich, St. Louis, USA. DMEM high glucose media, fetal bovine serum (FBS), and antibiotic/antimycotic solution, Gibco, Thermo Fisher Scientific USA. Antibodies were procured from Cell Signaling Technology, Danvers, USA, and Sigma-Aldrich, St. Louis, USA. Clarity ECL Substrate and Nitrocellulose membrane, Bio-Rad Laboratories, California, USA.

### 2.2 Yashtimadhu procurement, authentication, and extract preparation

Yashtimadhu choorna (Lot No.64), prepared from the roots of *Glycyrrhiza glabra L.* was procured from SDP Remedies and Research Centre, Puttur, Kaarnataka, India, a GMP-certified Ayurvedic product manufacturer (http://sdpayurveda.com/products/choorna/yastimadhu-choorna/). A specimen of it is maintained at the SDP Remedies and Research Centre (Identifier No. SDP/YM/001-2017). The industrial process includes the following steps; the roots of *G. glabra L.* were washed and dried under shade-net, followed by vacuum-drum drying. Dried roots were pulverized and sieved to yield the fine powder, giving a yield of about 90%.

For cell culture, Yashtimadhu root powder was suspended in MilliQ-water (0.1 g/mL) and incubated overnight at room temperature, with continuous rotation. The mixture was centrifuged at 5000 rpm for 10 min, twice. Aqueous supernatant was carefully aspirated into a new tube and dried using SpeedVac (Savant, Thermo Fisher Scientific, USA). The yield from the aqueous extraction of 1g of Yashtimadhu powder was found to be 54% w/w. The dried extract was dissolved in serum-free media for cell culture treatment.

### 2.3 Cell culture and treatment

IMR32 cells (ATCC® CCL-127™) were procured from National Centre for Cell Science, Pune, India. The cells were cultured in DMEM-high glucose medium supplemented with 10% FBS and antibiotic/antimycotic solution and maintained at 37°C with 5% CO_2_. Differentiation of IMR-32 cells was carried with DMEM-high glucose medium supplemented with 10 µM retinoic acid and 2% FBS for nine days. The expression of tyrosine hydroxylase was used to assess differentiation. Post-differentiation, the cells were treated with the following; 50 nM or 100 nM rotenone (dissolved in DMSO), 200 µg/mL Yashtimadhu extract, and co-treatment of rotenone and Yashtimadhu, for 48 h, while untreated cells were taken as control.

### 2.4 Cell cytotoxicity assay with rotenone and Yashtimadhu

IMR32 cells were seeded at 5000 cells/well, in a 96-well plate, and subject to treatment with different concentrations of rotenone (0.25 nM–10 µM) for 24h and 48h, Yashtimadhu (50–1500 µg/mL) for 48h and, co-treatment of Yashtimadhu and rotenone for 48h. MTT-dye was incubated for 3 -4 h; formazan crystals were dissolved using 50:50 ethanol: DMSO and read at 560 nm and 650 nm. Cell cytotoxicity is expressed as percentage cell viability with respect to untreated control. Cell cytotoxicity assay was conducted as three technical replicates and three independent biological replicates.

### 2.5 Staining of live and dead cells

Differentiated and treated cells (rotenone, Yashtimadhu, and rotenone + Yashtimadhu), were stained with 20 µg/mL of propidium iodide (PI), for dead cells and counter-stain, Hoechst-33342 (5 µg/mL) [9]. Cells were imaged using ZOE™ Fluorescent Cell Imager, BioRad Laboratories, California, USA. ImageJ software [10] was used to calculate the ratio of PI to Hoechst-33342 stained cells. The percentage of cell death was calculated with respect to control and later converted to fold change. The live-dead cell cycle was performed as two technical replicates and three independent biological replicates.

### 2.6 Analysis of cell cycle

Treated cells were washed with 1X PBS, trypsinized and further washed twice with 1X PBS and re-suspended in hypotonic buffer (2 µg/mL PI, 1 mg/mL trisodium citrate, 0.1% Triton-X 100 and, 100 µg/mL RNase), incubated in the dark for 30 min. The red fluorescence was measured using Guava® easyCyte Flow Cytometer, EMD Millipore, Massachusetts, USA. Cell cycle data analysis was carried out with FCS Express (version-6). The cell cycle data was performed as independent three biological replicates.

### 2.7 Immunoblotting

Harvested cells were lysed in buffer [4% sodium dodecyl sulfate in 50 mM triethylammonium bicarbonate, with sodium orthovanadate (1mM), sodium pyrophosphatase (2.5 mM), and, beta-glycerophosphate (1 mM)]. Following proteins were assessed, the primary antibody along with dilution factors are mentioned in brackets; cleaved-caspase-9 (1:1000), cleaved caspase-3 (1:1000), cleaved-Poly-ADP-ribose polymerase (PARP, 1:1000), BCL2-associated-X protein (BAX, 1:1000), BCL2-antagonist/killer-1 (BAK1, 1:1000), pyruvate dehydrogenase-E1 alpha-1 (PDHA1, 1:1000), cytochrome-C (1:1000), superoxide dismutase-1 (SOD1, 1:1000), heat-shock protein-60 (HSP60, 1:1000), phosphorylated extracellular-signal-regulated kinase-1/2 (ERK-1/2, 1:1000), total ERK1-/2 (1:1000) were procured from Cell Signaling Technology, USA and, HRP-conjugated β-actin (1:50000) was used a loading-control. Immunoblotting analysis of the above-mentioned proteins were carried out as three independent biological replicates.

Protein concentration estimated with BCA assay and immunoblotting was carried out as described previously [11]. Briefly, equal amounts of proteins from the four conditions were loaded onto SDS-PAGE and resolved. The resolution of proteins was carried out with SDS-PAGE, and the proteins were transferred onto a nitrocellulose membrane. Upon completion of the transfer, the membranes were blocked with 5% skimmed milk for 1h. Membranes were incubated in primary antibodies, which were diluted in 3% bovine serum albumin (BSA) overnight. Washing of membranes was carried out with 1X PBST (phosphate-buffered saline with 0.5% Tween-20) and further incubated with 1:5000 dilution HRP-conjugated secondary antibody in 3 % skimmed milk for 2h. The bands were imaged and developed with Enhanced Chemiluminescence reagent, BioRad, and captured onto X-ray films, Carestream. Densitometry analysis of band intensity was performed using ImageJ, normalized with loading-control, and fold-change calculated with respect to control. A heat map depicting the protein expression was prepared using Morpheus.

### 2.8 Protein-protein interaction

The proteins assessed with western blotting were further analyzed for their interaction network using ANAT (Advanced Network Analysis Tool) plug-in [12], in Cytoscape, version 3.8. The anchored network was built, and the significance of the interactions was obtained using the Gene Ontology (GO) and KEGG pathways using the ANAT tool, using the default settings for carrying out the protein-protein interaction network.

### 2.9 Statistical analysis

The data from the independent biological replicates were used for the statistical testing. GraphPad Prism 5 was used for the statistical analysis, using One-way ANOVA (Analysis of Variance) with Bonferroni-corrections to identify the significant pairs. The ANAT tool gives the *p-*value of enrichment, which was used for statistical significance. A *p-*value of ≤ 0.05 was considered statically significant.

## Ethics Statement

The data described here did not involve the use of human or animal studies.

## CRediT Author Statement

**Gayathree Karthikkeyan:** Methodology, Validation, Formal analysis, Investigation, Writing – original draft, Visualization; **Ashwini Prabhu:** Methodology, Investigation, Resources; **Ravishankar Pervaje:** Conceptualization, Resources; **Sameera Krishna Pervaje:** Formal analysis; **Prashant Kumar Modi:** Conceptualization, Methodology, Resources, Writing – review & editing, Supervision, Project administration, Funding acquisition; **Thottethodi Subrahmanya Keshava Prasad:** Conceptualization, Methodology, Resources, Writing – review & editing, Supervision, Project administration, Funding acquisition.

## Declaration of Competing Interest

The authors declare that they have no known competing financial interests or personal relationships which have, or could be perceived to have, influenced the work reported in this article.

## Appendix Supplementary materials

Image, application 1
